# Implementation of an evidence-based intervention to improve the wellbeing of people with dementia and their carers: study protocol for ‘Care of People with dementia in their Environments (COPE)’ in the Australian context

**DOI:** 10.1186/s12877-018-0790-7

**Published:** 2018-05-09

**Authors:** Lindy Clemson, Kate Laver, Yun-Hee Jeon, Tracy A Comans, Justin Scanlan, Miia Rahja, Jennifer Culph, Lee-Fay Low, Sally Day, Monica Cations, Maria Crotty, Susan Kurrle, Catherine Piersol, Laura N. Gitlin

**Affiliations:** 1 Faculty of Health Sciences, The University of Sydney, Centre of Excellence in Population Ageing Research, Sydney, 2006 Australia; 2 Flinders University, Adelaide, Australia; 3 The University of Queensland, Brisbane, Australia; 4 Thomas Jefferson University, Philadelphia, USA; 5 Dexel University, Philadelphia, USA

**Keywords:** Dementia, Implementation science, Caregiver, Nonpharmacological interventions, Functional decline, Occupational therapy, Nursing

## Abstract

**Background:**

There are effective non-pharmacological treatment programs that reduce functional disability and changed behaviours in people with dementia. However, these programs (such as the Care of People with dementia in their Environments (COPE) program) are not widely available. The primary aim of this study is to determine the strategies and processes that enable the COPE program to be implemented into existing dementia care services in Australia.

**Methods:**

This study uses a mixed methods approach to test an implementation strategy. The COPE intervention (up to ten consultations with an occupational therapist and up to two consultations with a nurse) will be implemented using a number of strategies including planning (such as developing and building relationships with dementia care community service providers), educating (training nurses and occupational therapists in how to apply the intervention), restructuring (organisations establishing referral systems; therapist commitment to provide COPE to five clients following training) and quality management (coaching, support, reminders and fidelity checks). Qualitative and quantitative data will contribute to understanding how COPE is adopted and implemented. Feasibility, fidelity, acceptability, uptake and service delivery contexts will be explored and a cost/benefit evaluation conducted. Client outcomes of activity engagement and caregiver wellbeing will be assessed in a pragmatic pre-post evaluation.

**Discussion:**

While interventions that promote independence and wellbeing are effective and highly valued by people with dementia and their carers, access to such programs is limited. Barriers to translation that have been previously identified are addressed in this study, including limited training opportunities and a lack of confidence in clinicians working with complex symptoms of dementia. A strength of the study is that it involves implementation within different types of existing services, such as government and private providers, so the study will provide useful guidance for further future rollout.

**Trial registration:**

16 February 2017; ACTRN12617000238370.

## Background

Functional decline is one of the core features of dementia [1, 2]. As the symptoms of dementia worsen, the person becomes increasingly dependent on others for assistance with activities of daily living. Decline in cognitive and physical function is associated with reduced quality of life in the person with dementia, considerable impact on carers, increased use of health and social care resources and often culminates in the need to move to residential care [3–5].

There is now evidence from multiple randomised controlled trials that functional decline can be delayed in people with dementia [6–11]. Moreover, non-pharmacological interventions that work with both the person with dementia and their carers (dyadic interventions) and include strategies to promote independence and manage symptoms are more effective than pharmacological agents [12] and do not have the associated side effects [13]. Dyadic interventions are associated with a range of other benefits including: reduced carer burden, anxiety and depression, improved carer knowledge, and delayed time to institutionalisation [14–16]. While the ingredients of interventions vary, research suggests that interventions that are tailored and involve multiple components (e.g. carer education plus skills training plus engaging the person with dementia in activities) are most effective [14].

Despite evidence in favour of dyadic interventions and public support for such programs [17], access is limited [18]. Most of the programs found to be effective in research trials have been tested outside of existing care systems therefore the feasibility of providing the programs in routine service delivery is unclear [15]. Implementation is the use of strategies to adopt and integrate evidence-based health interventions and change practice patterns within specific settings [19]. The need for improved translation of research into practice has been recognised by the World Health Organization who have called for implementation of evidence based interventions that enhance function and capability in people with dementia in their global action plan [20]. The plan also calls for more research to provide information about *how* to translate evidence based programs into action [20].

There are currently few examples of the implementation of evidence based interventions for community dwelling people with dementia and their carers into real-world settings [15, 21] and none of these have taken place in Australia. Of those that do exist, a modified version of the original evidence based treatment has been applied suggesting that some adaptations are required to enhance feasibility in translation [22, 23]. One such program, the Community Occupational Therapy in Dementia (COTiD) project in the Netherlands, involved looking at barriers and facilitators to delivering the intervention as perceived by occupational therapists who had received training in the intervention [24]. COTiD involves ten consultations with an occupational therapist delivered over a shorter time frame (five weeks) and tends to focus mostly on activities of daily living. Focus groups revealed that therapists did not feel competent in implementing the program, had difficulty providing the amount of treatment recommended in the intervention guideline and struggled with the structured nature of the intervention including the amount of documentation associated. Yet, they valued the resources provided within the program, were positive about the evidence supporting the program and benefited from support from their colleagues. Physicians and managers who were involved in the study reported a lack of awareness about the COTiD intervention and referral mechanisms to occupational therapists were not clear or easy to complete. An additional implementation project involving the COTiD intervention, which aimed to address these barriers and facilitators, involved training days, outreach visits, regional meetings and a web based discussion platform. The effectiveness of the implementation strategy was tested in a cluster randomised trial and process evaluation. Results of the study revealed that the referrals to the COTiD program could be increased but adherence to the intervention was not enhanced following the implementation strategies [23, 25].

A second program of implementation conducted in the United States involved implementation of the Environmental Skill-Building Program (ESP; renamed as Skills_2_Care^R^) within a homecare practice [22]. The implementation involved site preparation, training, establishing referral mechanisms and evaluation. A total of 22 therapists were trained to provide the intervention and provided an average of 4.7 sessions; the implementation was considered moderately successful. Fidelity to the intervention was variable and fidelity checks were difficult to conduct within the homecare organisations.

This study examines implementation of the ‘Care of People with dementia in their Environments’ (COPE) program in the Australian context [26]. COPE is a non-pharmacological intervention designed to reduce functional disability in people with dementia. The program comprises occupational therapy and nursing input (involving 8-10 consultations with an occupational therapist and two consultations with a nurse) delivered over four months. Core elements of the program include: focusing on the capabilities of the person with dementia, prevention and management of changed behaviours and carer support and education. Strategies applied by the therapist and nurse include carer education and strategies to modify communication, tasks and the environment. A large randomised trial (*n* = 237) conducted in the United States found that the program was effective in reducing dependency and increasing engagement of the person with dementia and improving carer wellbeing [26]. At four months carers reported significantly higher levels of wellbeing. At nine months carers in the intervention group reported a “great deal” of improvement in their lives overall, confidence managing changed behaviours and improved ability to keep living at home.

The main research questions for this project are:

1. How is COPE adopted, implemented and made sustainable within different community health contexts in Australia?
2. What are the costs associated with delivery of COPE and are there changes in resource utilisation of people with dementia before and after intervention, and

We will also conduct a pragmatic pre-post evaluation to investigate:

When implemented into existing services, does COPE have the same size of effect for activity engagement outcomes for the person with dementia and wellbeing outcomes for the carer as when tested in the randomised controlled trial?

## Methods

### Design

This implementation research project employs a mixed methods research design [27] to evaluate the process of implementation of the COPE project. According to Curran’s classification of effectiveness-implementation hybrid trials, the trial utilises a Type 3 design via testing an implementation strategy and collecting information on the clinical intervention and outcomes [28]. The study seeks to understand what, why and how the COPE intervention will work in the Australian setting within existing programs and resources. The mixed methods design [27] includes collection of qualitative and quantitative data from both health professionals employed by the partner organisations and the people receiving the program (people with dementia and their carers).

**Fig. 1:**
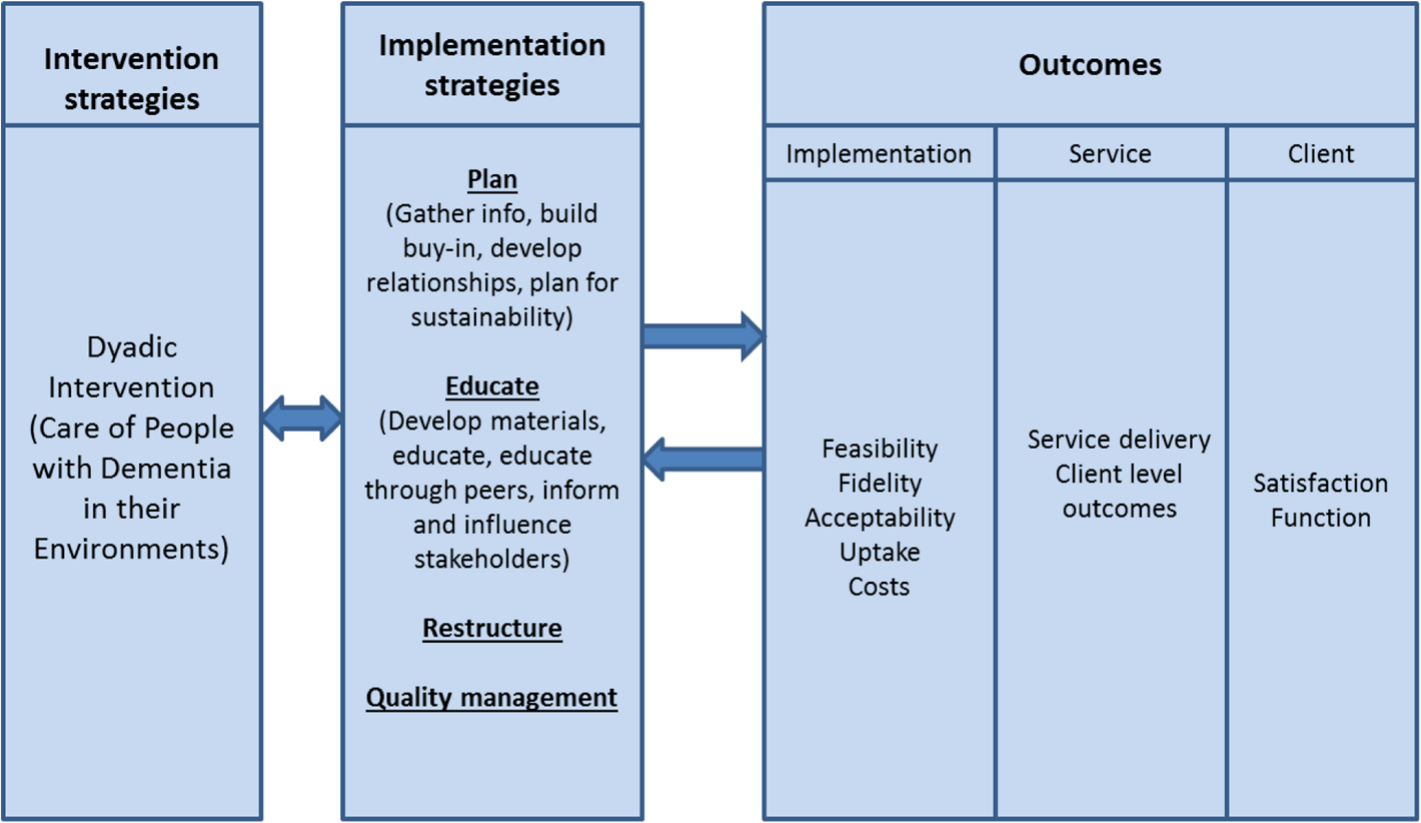
Implementation model used within the project. This figure depicts an overview of the project using the reporting format recommended by Proctor et al. [47]

An overview of the implementation model used within the project is presented in Fig. 1. The *intervention strategy* is the COPE program, an evidence based dyadic intervention, which has been described briefly above as well as in the original paper published by Gitlin and colleagues [26]. *The implementation strategy* involves a number of components including the development of relationships with existing organisations/service providers who provide services for people with dementia. These organisations include government services, not-for-profit aged care services, and private services; this will allow us to explore the settings in which implementation is most likely to work and will assist with sustainability and scaling up where implementation is successful. Implementation strategies were formed based on known relevant barriers and enablers which are described in Table 1 and possible solutions to these barriers designed by Michie and colleagues and as described in the behaviour change wheel [29].

**Table 1:** Barriers and enablers and possible solutions proposed within the behaviour change wheel [29]

Behaviour Change Wheel components	Barriers and enablers	Possible solutions
Capability	Health professionals have reported limited knowledge regarding best practice dementia care [33]. Working with people with dementia and carers can be complex and requires high level cognitive and interpersonal skills [23]. Therapists tend to provide care-based established patterns and changing therapist behviour is difficult [49].	Education, Training, Enablement
Opportunity	Evidence supports programs delivered over a number of consultations which can be difficult to achieve in the Australian settings [50]. Occupational therapists often receive referrals for home and safety assessments rather than management of the symptoms of dementia	Environmental restructuring, Enablement
Motivation	Therapists report reduced confidence in intervention so may lack confidence that they can make a difference [33]. Therapists tend to work autonomously so they receive little positive reinforcement about their work from within their organisation or from their peers.	Educate, Persuasion, Enablement

As portrayed in Fig. 1, our primary *outcome* is related to implementation but we will also measure outcomes at the level of the service and the client.

### Study setting and context

Most aged care services in Australia are funded by the state or federal government and are delivered either via a state government health service or a non-government organisation [30]. There are also services offered by private practitioners. Care may be provided for a short period or on an ongoing basis. Short term services include restorative care (early intervention to optimise function and independence) and transition programs (post hospital or illness) which are goal-oriented, time-limited and therapy focussed. Ongoing home care packages are to maintain independent living for as long as possible in one’s own home, subsidising a package of care, services and case management depending on need. People with dementia and their carers are also able to access helplines and advisory services which provide education about dementia and advice regarding managing changed behaviours ([31].

In summary, care of community-dwelling people with dementia in Australia is currently fragmented and services are provided in a number of different settings by a range of different health professionals [32]. While there are existing services that provide intervention and care for people with dementia and their carers in the community, care approaches tend to focus on assessment and case management and there are a lack of programs which offer evidence based non-pharmacological treatments to optimise independence and manage the symptoms of dementia [32, 33]. The study is based in two states of Australia: New South Wales (NSW) and South Australia (SA) providing an additional geographical perspective. We aim to recruit a mix of government services, non-government organisations and private practitioners within both of these states.

### Participants

#### Participating organisations

The research team will establish agreements with 10-20 different organisations or individual service providers where there is a fit between the project and the organisation/provider and this is supported by managerial staff. All organisations will provide services for community dwelling people with dementia and will employ occupational therapists. All organisations will either employ nurses or be able to link with community nursing services to provide the nursing component of the intervention. As stated, we will deliberately seek to establish agreements with a mix of government and non-government organisations as well as private occupational therapy services in order to examine adoption in different contexts. Each participating organisation will be asked to nominate a team who will be key players in the implementation of COPE comprising someone in a management position, an occupational therapist and a nurse.

#### Participating occupational therapists and nurses

Occupational therapists and nurses who work at the participating organisations will be nominated to be involved in the study by their participating organisation. These staff will have an existing caseload which includes people with dementia. Participating occupational therapists and nurses will attend training in the intervention and will be asked to consent five dyads (people with dementia and their carer) to participate in the research project. They will deliver the intervention with these five dyads and provide data on these clients for the purposes of the project. Therapists will be provided with certification after attendance at training and upon completion of the program with three dyads. Occupational therapists and nurses will be supported through mentoring and coaching sessions. We will recruit at least 30 therapists which provides considerable allowance for dropout and other circumstances which may prevent therapists from providing the COPE program to five dyads.

#### Participating people with dementia and their carers (dyads)

The 103 dyads involved in this study will be clients of the participating organisations and more specifically, clients of the participating occupational therapists and nurses. These participants will be identified as having the potential to benefit from the COPE intervention by the participating occupational therapists. Strict inclusion criteria will not be applied but therapists will be made aware of the target client group for whom the intervention was designed: People with a diagnosis of dementia (or probable dementia) or a Mini Mental State Examination score of less than 24 who need help with daily activities and/or have changed behaviours. The participants will live with or nearby someone who takes on a ‘carer’ role and the carer must report some difficulty in managing symptoms. The client’s own therapist will seek their consent to participate in the research study. Whether or not they consent to participate will not impact on the person’s ability to access the COPE program or occupational therapy intervention as part of their usual treatment. Participants deemed to be unable to consent will still be included in discussion about the study and verbal assent will be obtained as well as proxy consent.

**Fig. 2:**
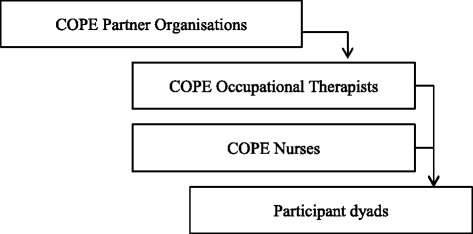
Study participants

A diagram of the relationship between the participating organisations, participating occupational therapists and nurses and participating people with dementia and their carers is presented in Fig. 2.

### Implementation strategies

**Fig. 3:**
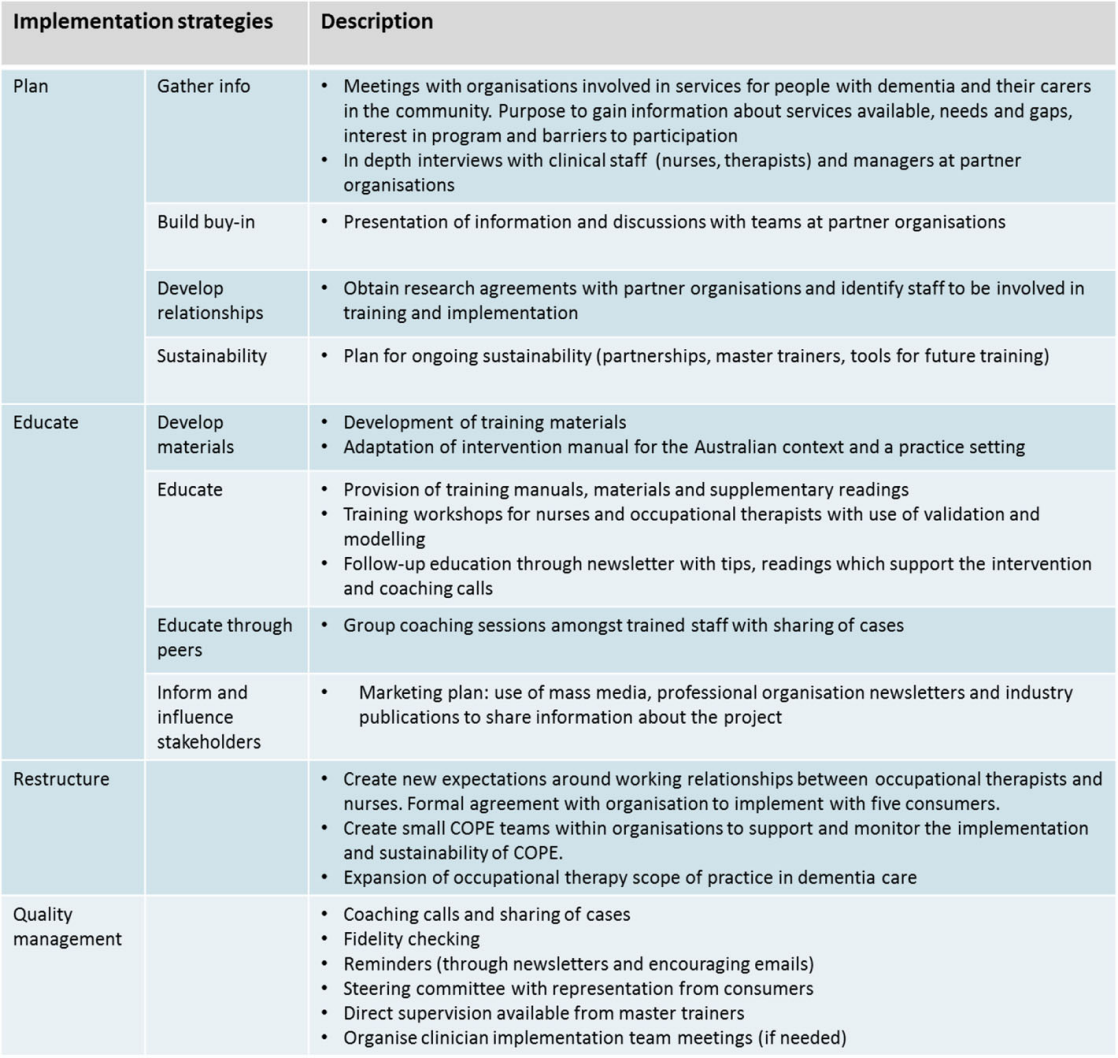
Description of implementation strategies used presented using the framework devised by Powell et al. [48]

We will draw on a number of implementation strategies including planning, education, restructuring, and quality management (presented in Fig. 3). Implementation strategies used within the project are guided by barriers and enablers and proposed solutions suggested using the behaviour change wheel.

#### Plan

This phase involves establishing relationships with participating organisations and exploration of preparation for change within these organisations. This process will involve sharing of information in order to understand the scope and work of COPE and how this may fit with the implementation of COPE within their organisation and the role of the research team. Discussions with managers and potential interventionists will enable identification of common work, values and goals. This is an iterative process and the ongoing relationship will influence identification of other support strategies to be shared and enacted by the researcher/organisation partnership. Planning for sustainability will be encouraged from the beginning through collection of data to contribute to a business case, formalizing partnerships (with enough partners involved to sustain changes if some withdraw), creation of master trainers and tools and materials for ongoing planning as well as wide promotion of the program for a diverse group of audiences which will raise awareness of the program.

#### Educate

We will work closely with the primary developers of the COPE program (including author’s LNG & CP), establishing the mutable and immutable aspects of the program. Training and manuals originally designed for use in research trials in the United States will be refined for the Australian context to ensure cultural appropriateness. A member of the research team will pilot the modified program with five dyads to ensure applicability to the Australian context and seek informal feedback regarding the utility and content of the program.

Participating occupational therapists and nurses will attend training to understand the theory and application of the intervention. Clinicians will be provided with the intervention manual, documentation and associated resources and taught ‘what to do’ and ‘how to do it’. They will be taught the program structure and content as designed by the original developers of the program (with modifications for the Australian context). Training in delivering the intervention will be provided over two days for occupational therapists and approximately two hours for nursing staff. Training methods have been deliberately designed to build self-efficacy in clinicians by: (a) facilitating mastery through experience (role play), (b) hearing descriptions of scenarios in which the program has been delivered and had good results (modelling), (c) hearing descriptions of the supporting evidence and ability for therapists and nurses to make a difference to facilitate belief in the intervention and (d) positive feedback and validation. Clinicians will be informed that the training enables them to deliver the intervention as developed by the team in the US but that in practice, they may need to make some amendments so that the intervention fits within their existing role and resources (for example where they are unable to provide services over four months). We will work with our participating organisations to promote the implementation work within the media, professional organisations and local newsletters or newspapers. The goal of this promotional work is to inform and influence stakeholders (both those who are already participating and other related organisations).

Barriers and enablers to implementation which are identified by the participating health professionals during the implementation phase will be noted and addressed. For example, if clinicians identify that they have trouble explaining the intervention to their colleagues or clients we can provide educational leaflets and strategies to assist with this.

#### Restructure

Participation in the implementation project involves therapists and nurses delivering the program to five or more dyads. This ensures that the therapist completes the training with the expectation that changes to practice will occur. Furthermore, the expectation that the occupational therapist and nurse will work together to deliver the program may provide the opportunity to create new working relationships in some organisations.

#### Quality management

Following the training, clinicians will receive support through regular, small group coaching calls (with their peers and a member of the research team) and direct mentoring or supervision if sought. The coaching calls provide the opportunity to share case scenarios and learn from the trainers and their peers. For the 12 months following training we will also provide support in the form of encouragement, reminders and newsletters. If needed, we will visit the site and meet with staff responsible for implementation as well as other people within the organisation who can provide support for implementation, such as managerial staff.

### Evaluation

The primary outcome of interest relates to the implementation of the intervention which will be measured in terms of feasibility, fidelity, acceptability, uptake and costs. Evaluation will also determine the efficacy of the COPE intervention when provided by participating organisations. We will assess whether there are beneficial outcomes for the person with dementia and their carer by comparing pre and post measures and then comparing any changes (by effect size) to the effect sizes reported in the original research trial We will also examine similarities and differences within the different service delivery contexts.

**Fig. 4:**
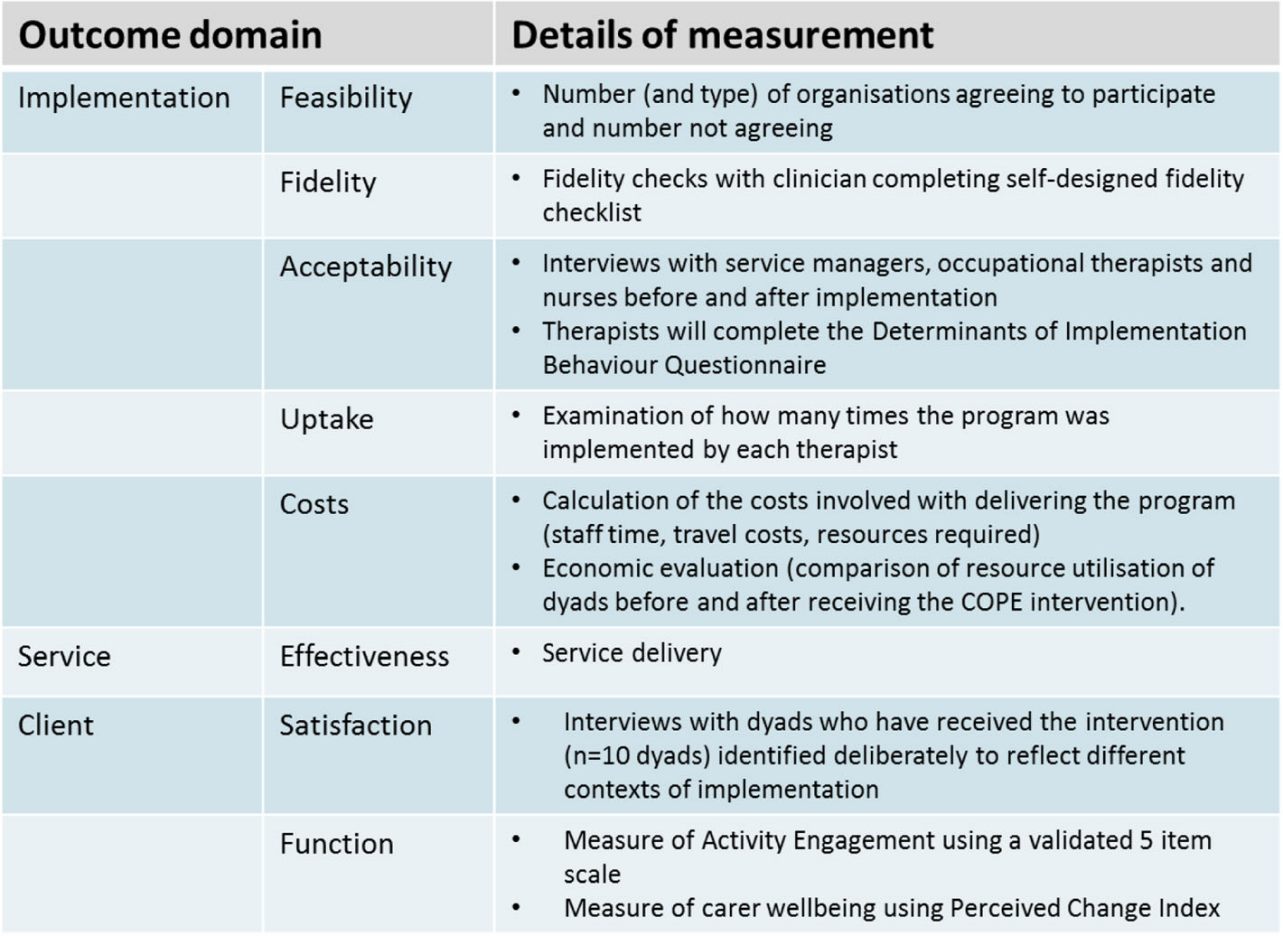
Outcomes assessed

Outcomes assessed within the project are presented in Fig. 4.

#### Implementation

Implementation will be measured by considering the number and type of organisations agreeing to participate. We will also assess uptake of the intervention over time; this information will be recorded by therapists who will provide information regarding on how many occasions they delivered the intervention. In addition, we will chart the duration between attending training and commencing implementation. Interviews with health professionals will be repeated after implementation of COPE and will provide information about acceptability from their perspective. These interviews aim to understand their experience of COPE, perceptions of change (or not) and factors that may have influenced uptake. Normalisation Process Theory can be seen as a framework to identify factors that encourage or impede the implementation of complex interventions. Normalisation Process Theory will be used as a tool in developing the interview guide, as it highlights the work that individuals do to incorporate innovation into the context of their organisational constructs and culture [34]. This information will be supplemented with information from the Determinants of Implementation Behaviour Questionnaire [35] which was developed to measure the behavioural determinants of implementation. The Questionnaire is based on the Theoretical Domains Framework and includes 93 items covering 18 domains including knowledge, skills and social/professional role and identity. Respondents are asked to what degree they agree or disagree with statements such as ‘I have the skills to deliver COPE following the guidelines’.

Uptake will also be assessed via a fidelity checklist completed by the occupational therapist providing the intervention. The fidelity check was designed specifically for this study. Fidelity will be assessed primarily in terms of adherence to the duration and content of the intervention provided and how this relates to the duration and content of the original trial. In addition to the number and length of sessions and the assessments conducted therapists are asked to record information about collaboration with the carer, how well the carer was able to use strategies and the perceived level of carer engagement.

Following establishment of the research agreement between participating organisations and the University of Sydney, participating organisations will be asked to describe the structure of their services including completion of a network map of their organisation. Network maps are useful in examining variables within a context and the complex interactions between them, which may be difficult to describe in an interview [36]. The aim is to develop an understanding about the strength of relationships between different people in the organisation, as well as the perceived source of innovation. We will also conduct in-depth interviews with up to 30 occupational therapists, nurses and service managers within the participating organisations to identify current practices, understand their beliefs about their own capabilities, skills and motivation, gain insight into their organisational culture and structures, explore their previous experiences with innovation and identify the predicted barriers and enablers to the implementation of COPE.

### Service: effectiveness, service delivery

Client: We will assess whether outcomes for the carer and person with dementia who received COPE within the study (participating dyads) are similar in terms of effect sizes as those that were demonstrated in the original randomised controlled trial. This evaluation will be a pragmatic pre-post intervention evaluation. Outcomes assessed include activity engagement of the person with dementia using a validated five-item scale in which carers are asked to rate the carer’s engagement in leisure and recreational activities from 1 (never) to 3 (often). We will also measure carer wellbeing using the 13 item Perceived Change Index in which carers are asked to rate changes in their wellbeing and coping over the last month. The pre-and-post-intervention dyad questionnaires include a measure of engagement of the person with dementia (a validated five-item scale which demonstrated an effect size of 0.26 (Cohen d) in the earlier trial [26]) and a measure of carer wellbeing, the Perceived Change Index (which had an effect size of 0.30).

Ten of the dyads who have received the program (who will be purposefully identified to represent different settings) will also be asked to participate in an interview. These dyads will be chosen using purposeful sampling, designed to reduce selection bias, and in keeping with the qualitative nature of the inquiry. During this interview, they will be asked a range of questions about how they are managing, how much help they need, and how confident the caregiver feels about providing that care.

### Analysis

#### Quantitative analysis

All quantitative data will be entered into SPSS. Outcome measures relating to impact on outcomes for the person with dementia and carer (engagement and carer wellbeing) will be analysed descriptively. Effect sizes will be calculated and presented using Cohen’s d and these will be compared to the effect sizes achieved in the original randomised trial of COPE. We calculate the estimated effect size of 0.26 will give a power of 80% (alpha error probability of.05) testing mean differences of time points using G-power (version 3) software which gave a sample of 93 dyads (before adding the dropout estimate). This is sufficient as the estimate for the 0.30 effect size is 71 dyads. We will recruit a total of 103 to allow for drop out.

A social cost benefit analysis (CBA) will be conducted to synthesise the costs and benefits of including COPE in the existing Australian health context. The CBA framework allows the identification of who bears the cost and who gains from the COPE program from multiple perspectives [37]. Monetary value will be assigned for all costs and outcomes using an established methodology [38] and the net social benefits of the intervention will be calculated. A positive overall net benefit represents an economic gain where theoretically the gainers could compensate the payers and still be better off. Probabilistic sensitivity analysis will be conducted to further interrogate the likelihood of COPE providing a net benefit to healthcare consumers, service providers and the Australian healthcare sector. To inform the analysis, costs relating to the provision of the COPE intervention will be collected. Direct costs include staff time in delivering the interventions (recorded in minutes), travel costs, and the cost of any resources provided (e.g. leaflets, equipment). Therapists providing the intervention will be asked to complete a form for each client which records the information necessary to calculate these costs. Resources will be costed at 2016 prices using actual cost of materials and current award wage rates. All dyads will be asked to complete the Resource Utilisation in Dementia (Lite) questionnaire [39] pre and post intervention. This will provide information on formal and informal care resources which can be used to value the care received.

#### Qualitative analysis

Interviews with staff and dyads will be audio-recorded, transcribed verbatim and entered into QSR NVivo. Thematic analysis (developing codes) will identify patterns within the study group [40]. A combination of inductive and deductive coding will be used. For participant dyads coding will commence with experience of COPE program and perceptions of change, but will be open to unexpected findings that may contribute to these. For health professionals, frameworks focusing on implementation and organisational culture will assist to synthesise the data gathered in order to build a comprehensive assessment of the barriers and facilitators; and thus informing implementation.

## Discussion

Implementation research is the scientific study of methods to promote the integration of research findings and evidence-based interventions into healthcare practice and policy [19]. The importance of implementation science is that it can accelerate the translation of effective interventions. This project is novel in that it is underpinned by theory and includes a broad framework approach that has enabled a focus on multi-component strategies that would best leverage implementation across a range of levels and practice settings, and utilises an iterative mixed method approach to understand the processes, context and complexity of changing practice. We seek to understand at the level of client, occupational therapist, nurse, manager and organisation and contribute to the knowledge base of how evidence-based interventions can be transported to real-world practice settings.

Evidence in favour of dyadic interventions is accumulating. Such interventions have the potential to delay functional decline, reduce carer impact, increase carer knowledge, reduce carer anxiety, reduce carer depression and delay time to institutionalisation [14–16]. Yet, implementation into routine practice has been poor. Surveys suggest that occupational therapists, who could provide these dyadic interventions, tend to focus on assessment and lack confidence in treating people with the symptoms associated with dementia [33]. This research project evaluates the process of implementation of the COPE intervention into a range of different service delivery contexts in Australia.

Strength of this study is its reach to three different types of practice settings which will enable comparison of differences and similarities within and between them. This project confirms the importance of attention to the local context, the engagement of stakeholder organisations, health care delivery settings and the role of individuals in dissemination and implementation [41]. It demonstrates that researchers and stakeholders need to work in partnership, develop working relationships and researchers to be attentive to need and context at individual and organisational levels. We know that elements such as ‘packaging’ the intervention through development of training, identifying core elements and skills training along with preparation for sustainability are important ([42, 43]) but the kinds of strategies and processes to achieve these are still evolving. In the case of dementia care in the community implementation will require a shift from ‘assessment’ to ‘intervention’ focused practice [15, 33]. It will also need to bridge the gap between the potential of empirically proven re-ablement programs, supported in current commonwealth aged care policies [44], to achieve their research aims in real-world settings. This project will provide information about how organisations fit these programs into the funding models they can already access. The extent that this project will impact on policy at the level of organisation, referral pathways and changing landscapes of access to re-ablement programs remains to be seen.

There will be future opportunities to compare cross-cultural implementation issues with another COPE study currently being undertaken in the US [45]. People with dementia who receive services through the Connecticut Home Care Program for Elders will be randomly allocated to receive COPE or usual care. The study aims to look at outcomes for the person with dementia and carer as well as net financial benefit, feasibility and acceptability when delivered within that home care program.

Our implementation study will provide detailed information about the process and outcomes of translation into Australian health contexts with rich qualitative data which will provide understanding about factors influencing implementation. Examining implementation in a range of settings and contexts will help inform the best models of fitting such programs within existing services. Further, challenges in scaling and building sustainability from early stages have received little attention [46]. Learnings from the study will outline strategies and processes for implementation and sustainability and we will better understand how establishing links with policy makers can support ongoing program delivery.

## Abbreviations

CBA: Cost benefit analysis
COPE: Care of People with dementia in their Environments
COTiD: Community Occupational Therapy in Dementia
